# Supplementary material for: Deletion of wfs1 Impairs Oligodendrocyte Precursor Cells Dorsal Distribution and Myelination Through the wfs1-hmgcs1 Axis in Zebrafish
Source: Biology (Basel). 2026 Aug 16;15(16):1402. doi: 10.3390/biology15161402 (PMC13510151; doi:10.3390/biology15161402)
Supplement: Supplementary file 1 [file biology-15-01402-s001.zip › File S1-Transcriptome sequencing data and differentially expressed genes (DEGs) in wfs1-- zebrafish at 5 dpf/2.KEGG/wfs1KOvsWT/DOWN/src/dre04142.html]

dre04142


- dre:571066

- Down regulated genes

571066(-7.1936807901335)

- dre:406511

- Down regulated genes

406511(-1.07481122268925)

- dre:565246

- Down regulated genes

565246(-2.15380681025991)

- dre:337771

- Down regulated genes

337771(-1.26044614136241)
- dre:445244

- Down regulated genes

445244\_1(-1.27471708253071)

- dre:337771

- Down regulated genes

337771(-1.26044614136241)
- dre:445244

- Down regulated genes

445244\_1(-1.27471708253071)

- dre:436725

- Down regulated genes

436725(-2.29340566555652)

- dre:553423

- Down regulated genes

553423\_1(-1.89310687503167)

Close
